# Supplementary material for: PD-L1+ plasma cells suppress T lymphocyte responses in patients with sepsis and mouse sepsis models
Source: Nat Commun. 2025 Mar 28;16:3030. doi: 10.1038/s41467-025-57706-9 (PMC11953283; doi:10.1038/s41467-025-57706-9)
Supplement: Supplementary file 1 — Supplementary information [file 41467_2025_57706_MOESM1_ESM.pdf]

PD-L1+ plasma cells suppress T lymphocyte responses in patients with sepsis and mouse sepsis models

Authors: Morgane Gossez[1,2], Clara Vigneron[2], Alexandra Vandermoeten[3], Margot Lepage[1,2], Louise Courcol[2], Remy Coudereau[1,4], Helena Paidassai[2], Laurent Jallades[2,5], Jonathan Lopez[6], Khalil Kandara[1], Marine Ortillon[1], Marine Mommert[4], Astrid Fabri[1], Estelle Peronnet[4], Clémence Grosjean[4], Marielle Buisson[7], Anne-Claire Lukaszewicz[4,8], Thomas Rimmelé[4,8], Laurent Argaud[9], Martin Cour[9], Bénédicte F Py[2], Olivier Thaunat[2], Thierry Defrance[2#], Guillaume Monneret[1,4#], Fabienne Venet*[1,2] - REALISM and RICO study groups

Supplementary Material

Supplementary Figure 1. Innate immune cells in spleen of Sham and CLP mice.

(A) Absolute counts of splenocytes (n = 23 Sham and 25 CLP mice). (B) Proportions among splenocytes and absolute counts of dendritic cells in 7 Sham and 12 CLP mice. (C) Proportions among splenocytes and absolute counts of NK cells in 13 Sham and 13 CLP mice. (D) Proportions among splenocytes and absolute counts of CD11b+ myeloid cells in 10 Sham and 13 CLP mice. (E) Proportions among CD11b+ myeloid cells and absolute counts of monocytes/macrophages in 10 Sham and 13 CLP mice. (F) Proportions among CD11b+ myeloid cells and absolute counts of neutrophils in 10 Sham and 13 CLP mice.

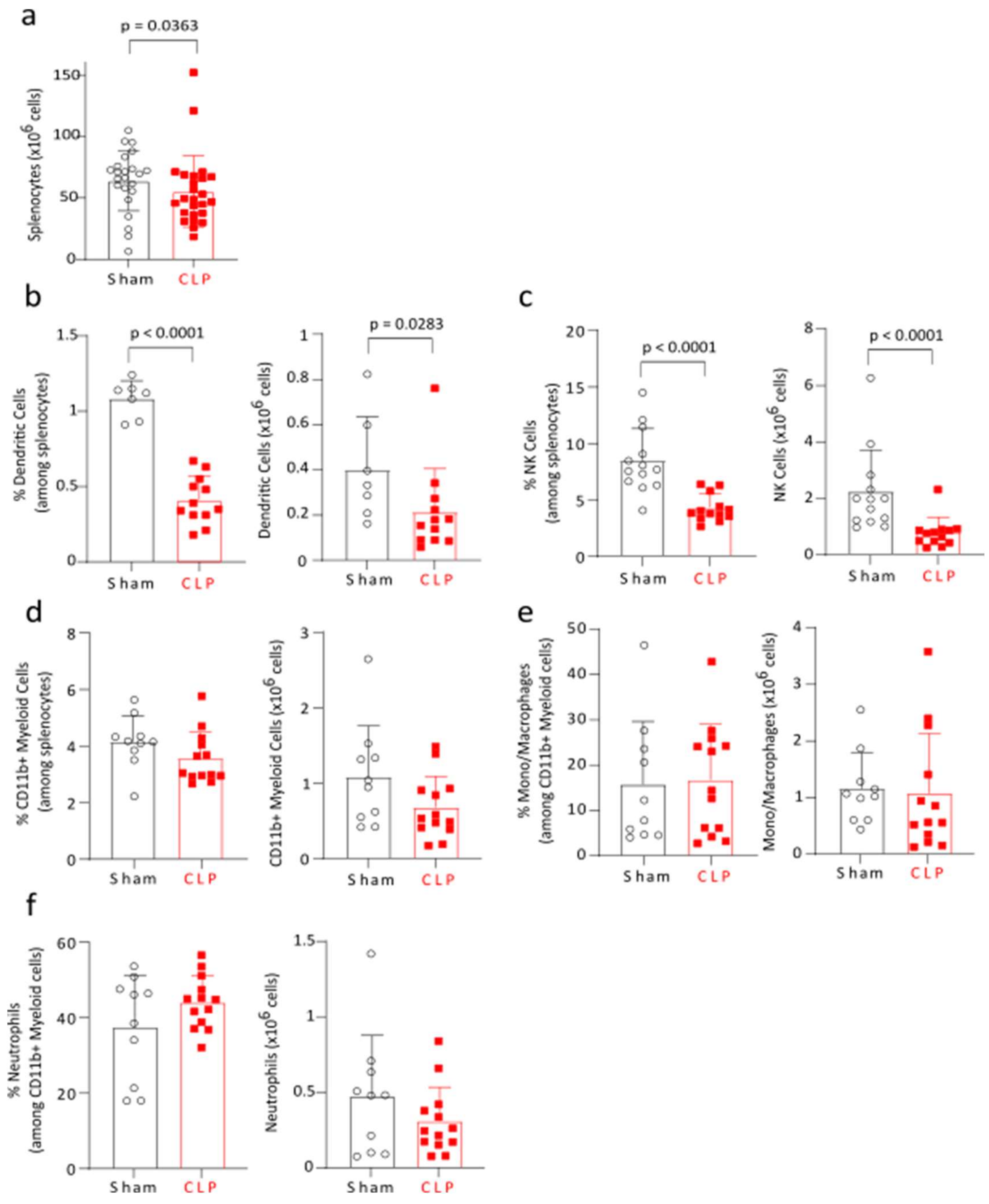

Supplementary Figure 2. T lymphocyte subpopulations in spleen of Sham and CLP mice.

(A) Proportions of CD4+ and CD8+ T cells among splenocytes in 23 Sham and 25 CLP. (B-D) Proportions among CD4+ T cells and absolute counts of naïve, central memory and effector CD4+ T lymphocytes in 11 Sham and 13 CLP mice. (E-G) Proportions among CD8+ T cells and absolute counts of naïve, central memory and effector CD8+ T lymphocytes in 11 Sham and 13 CLP mice (H) Absolute counts of CD4+CD25+CD127low regulatory T cell (n = 23 Sham and 25 CLP mice).

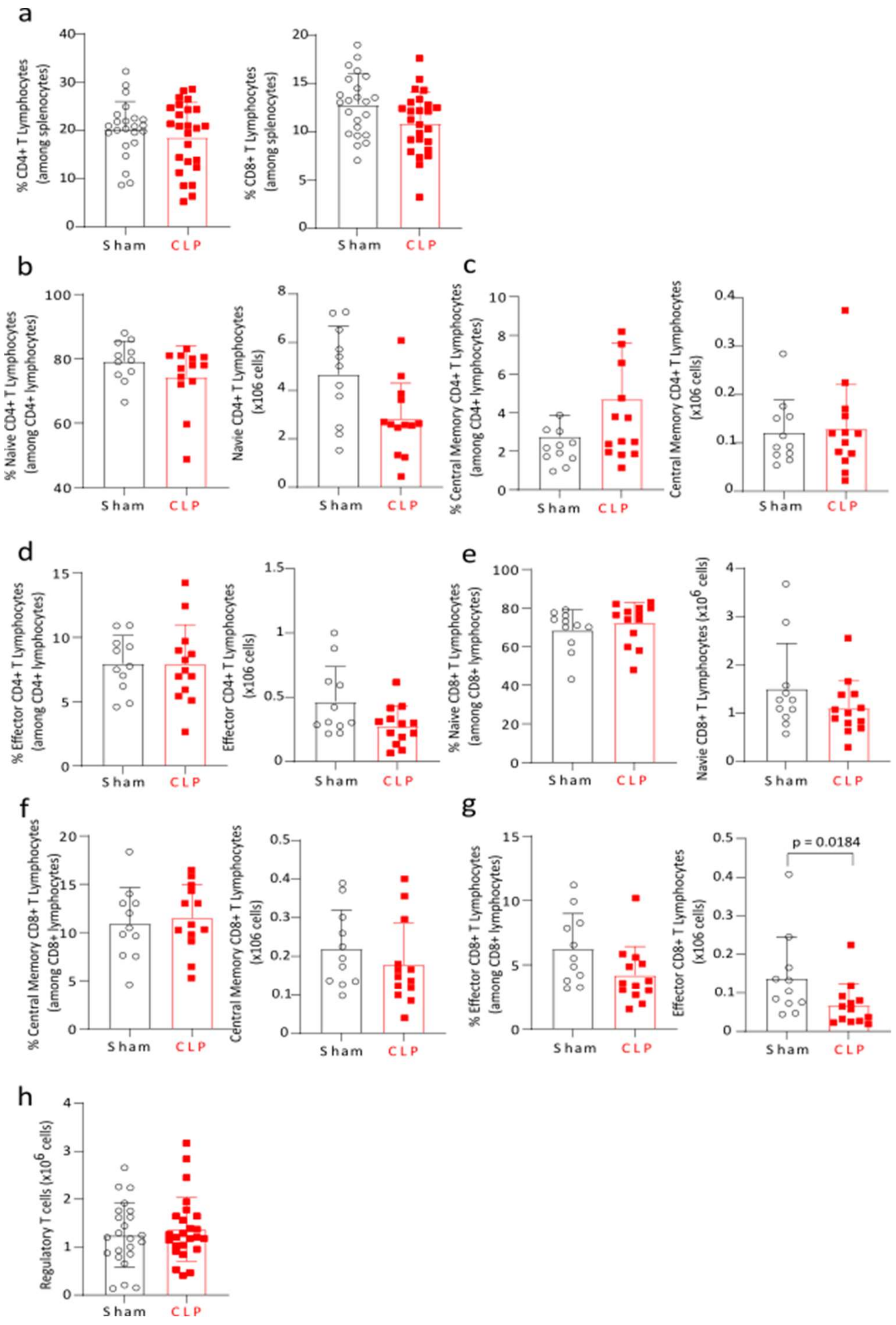

Supplementary Figure 3. Proliferation capacity of splenic T cells from Sham and CLP mice.

(A-B) Splenocytes from 5 Sham and 5 CLP mice were stimulated *ex vivo* by anti-CD3/CD28 Abs-coated beads. Cell proliferation was measured among viable cells by flow cytometry. Percentages of proliferating CD4+ (A) and CD8+ (B) T cells were evaluated. (C) Splenocytes from 5 Sham and 5 CLP mice were stimulated *ex vivo* by phytohemagglutinin. Cell proliferation was measured among viable cells by flow cytometry. Total percentages of proliferating CD3+ T cell and percentages of proliferating CD3+ T cell undergoing more than 3 or 4 division cycles were evaluated. Red squares represent results in CLP mice and white circles in Sham animals. Geometric means with standard deviations (SD) and individual values are shown. Mann-Whitney tests were performed and only significant p values < 0.05 are shown. (D) Splenocytes from ovalbumin-immunized mice were cultured with ovalbumin peptide at increasing concentrations (2, 5, 20 µg/mL). Percentages of IFNγ producing CD4+ T and CD8+ T were measured after 4h (n = 5 Sham and n = 5 CLP).

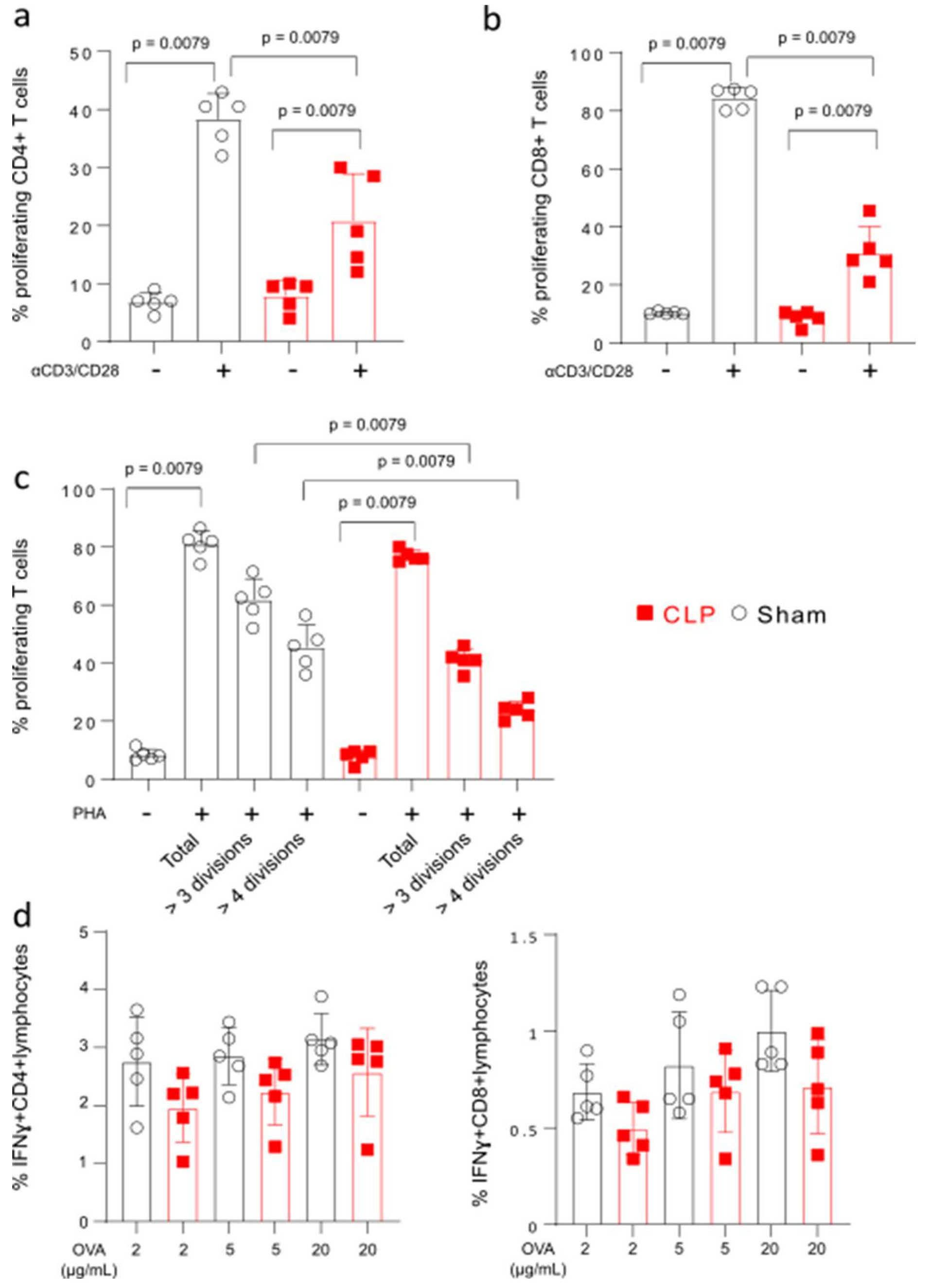

Supplementary Figure 4. Impact of the addition of B cells and plasma cells from septic patients in T cell co-culture assay.

Purified T cells from blood of healthy donors (n = 8) were cultured *ex vivo* in the absence or in the presence of stimulating anti-CD2/CD3/CD28 antibodies-coated beads, either alone or with B cells (n = 6) or plasma cells (n = 8) from septic patients at 1:1 ratio in the stimulated condition. Proportions of proliferating CD4+ (A) and CD8+ (B) T cells were measured by flow cytometry among viable CD4+ and CD8+ T cells respectively. Box-plots and individual values are shown. Non-parametric Wilcoxon paired tests were performed to compare results from different co-culture conditions. Only significant p values < 0.05 are shown.

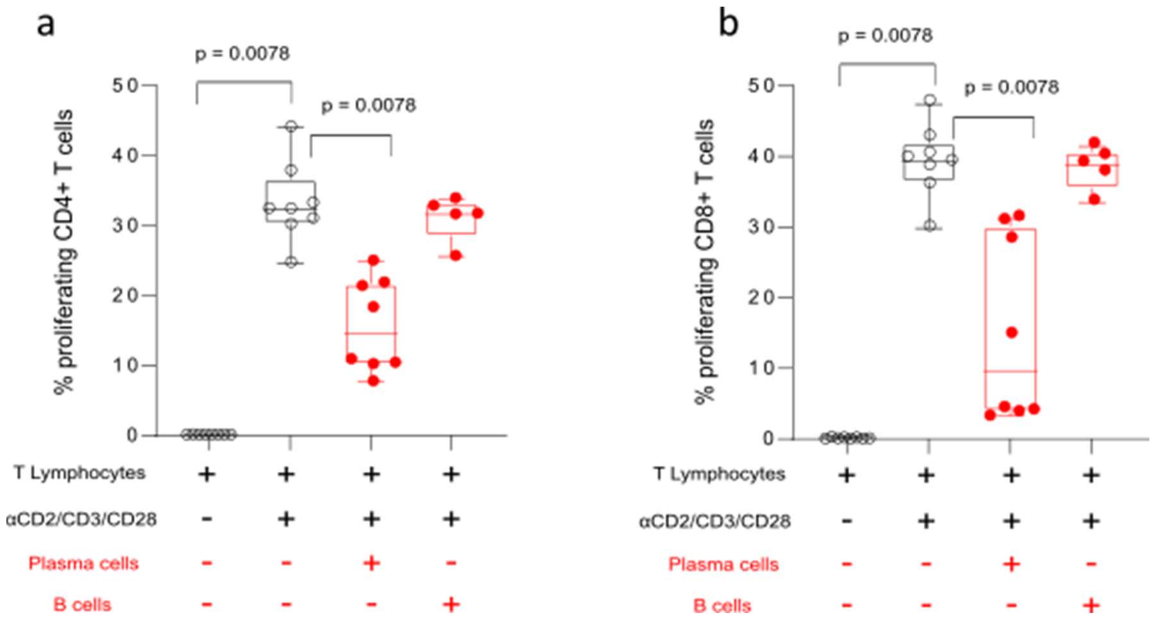

Supplementary Figure 5. Flow cytometry gating strategy and representative examples in mice for apoptosis and necrosis stainings on CD4+ and CD8+ T cells.

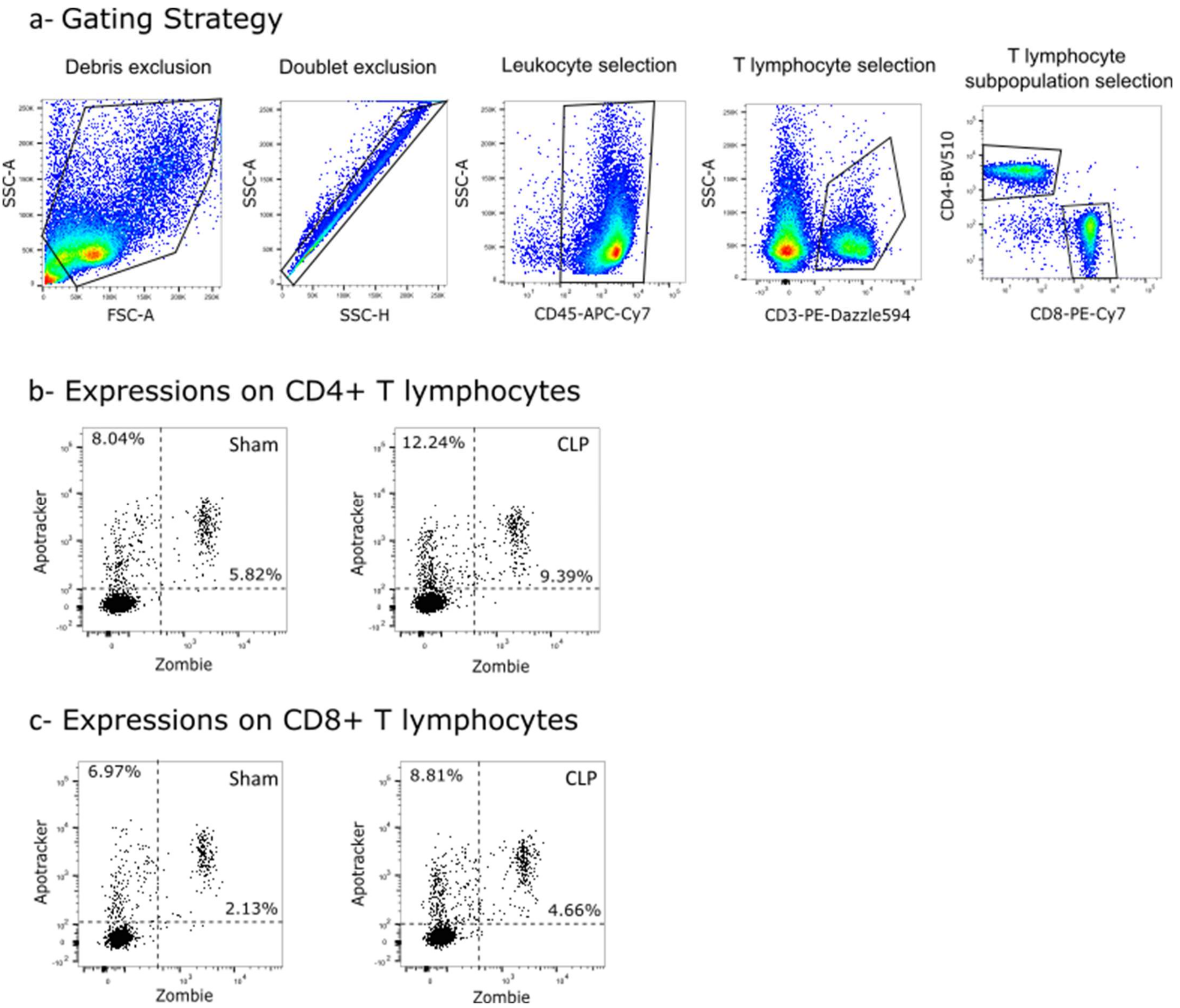

Supplementary Figure 6. Flow cytometry gating strategy and representative examples in mice for CD69 and PD-1 stainings on CD4+ and CD8+ T cells.

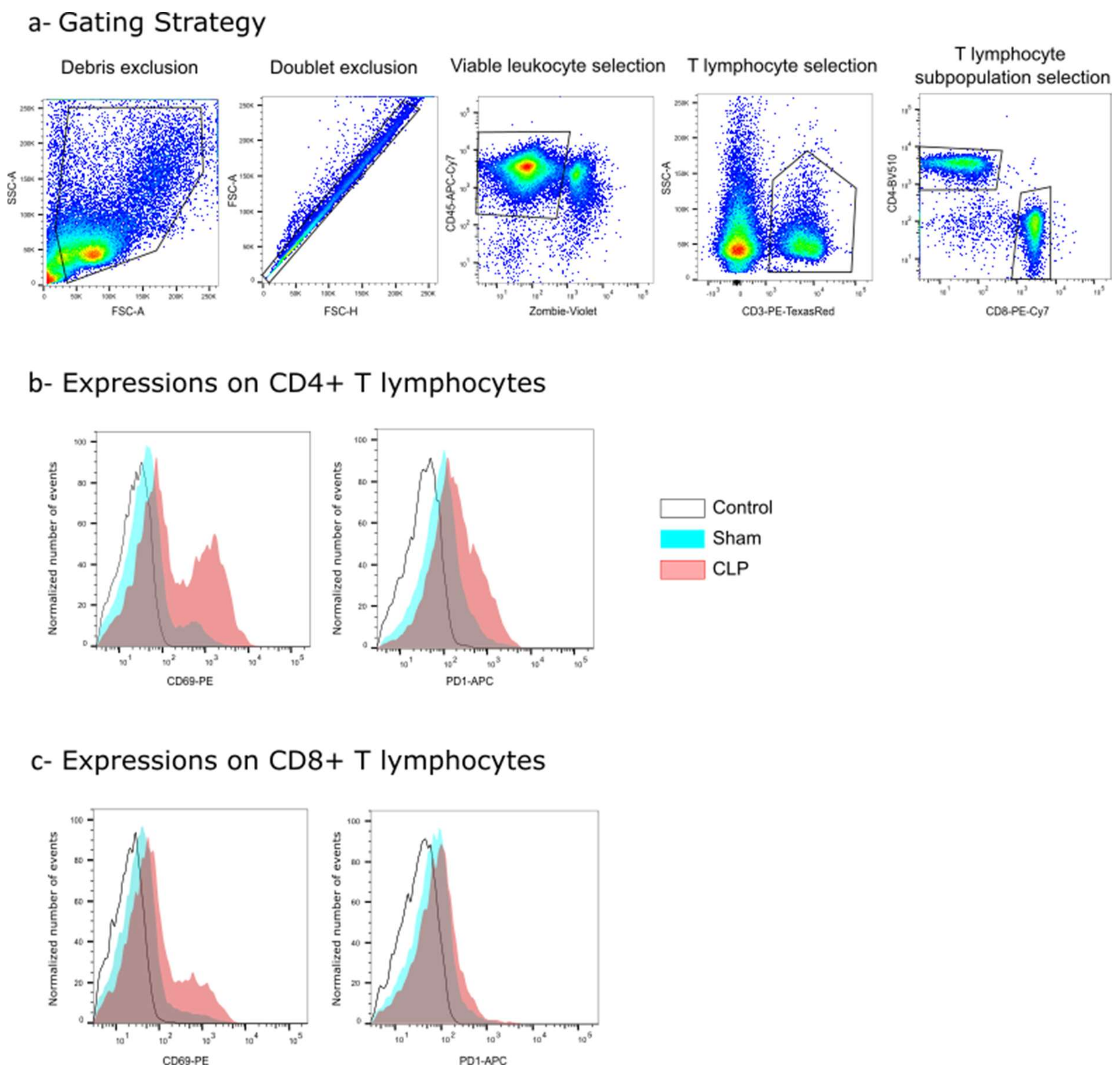

Supplementary Figure 7. Flow cytometry gating strategy and representative examples in mice for cell surface stainings on B cells and plasma cells.

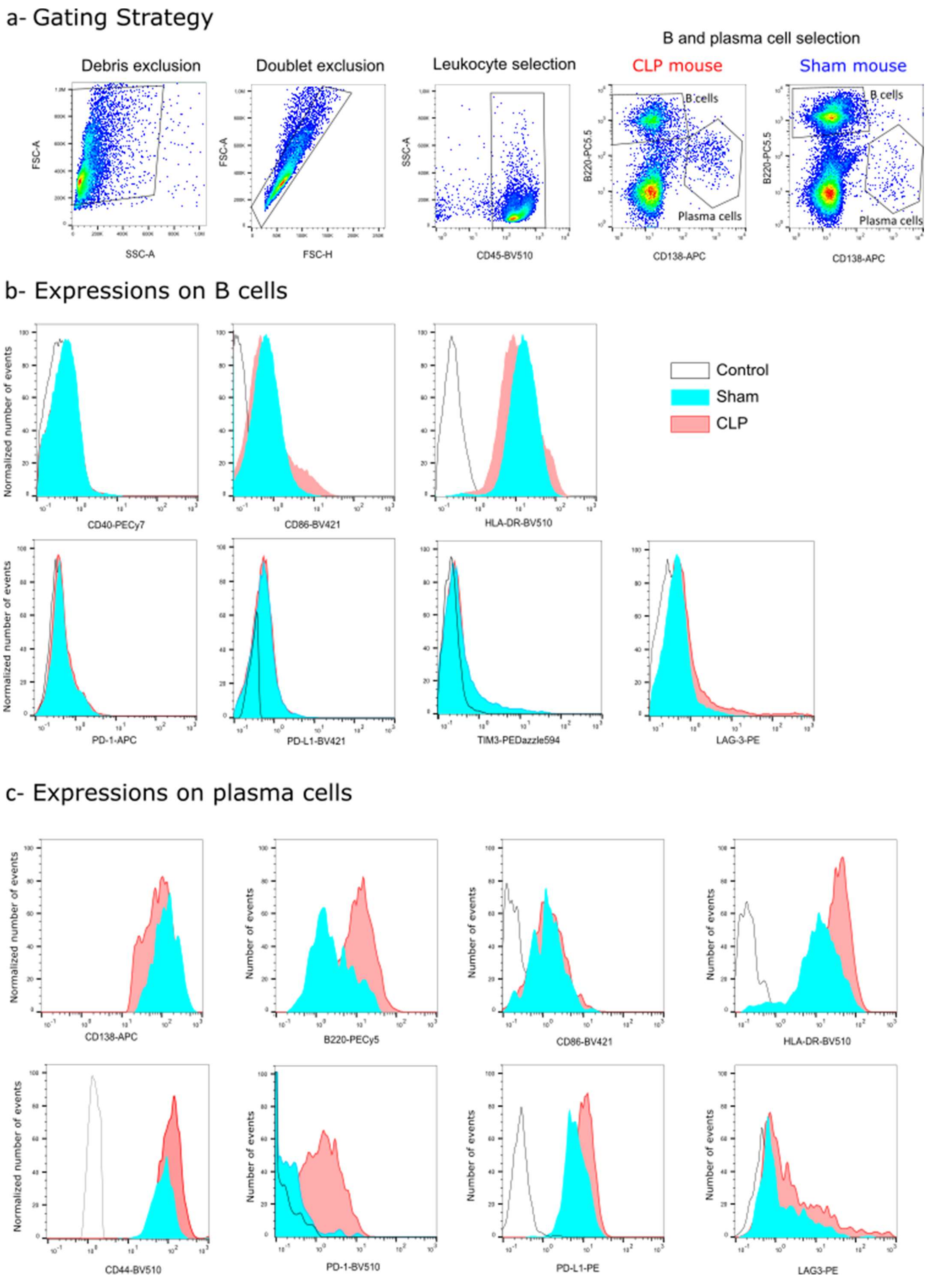

Supplementary Figure 8. Flow cytometry gating strategy and representative example of BLIMP-1 intracellular stainings in B cells and plasma cells from one CLP mouse.

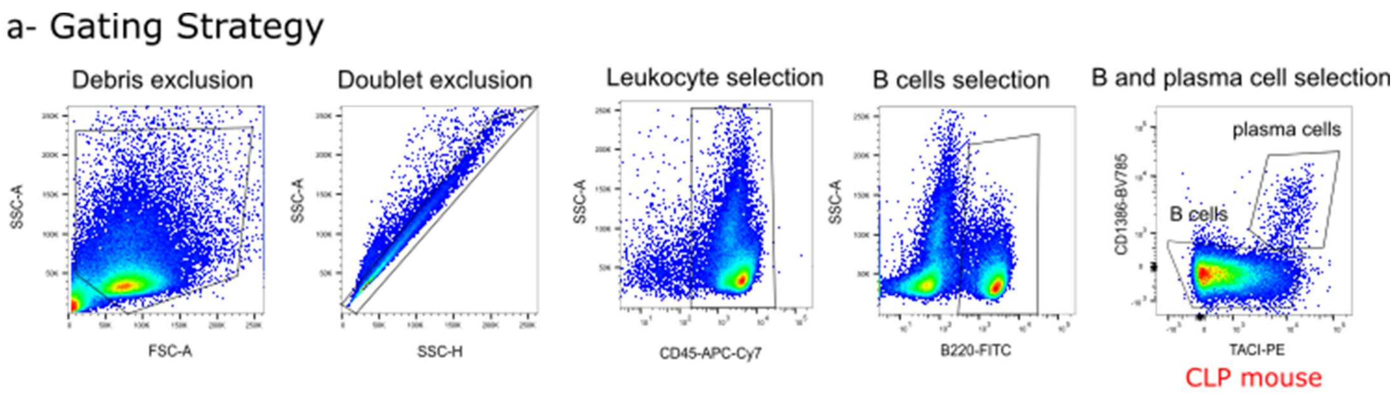

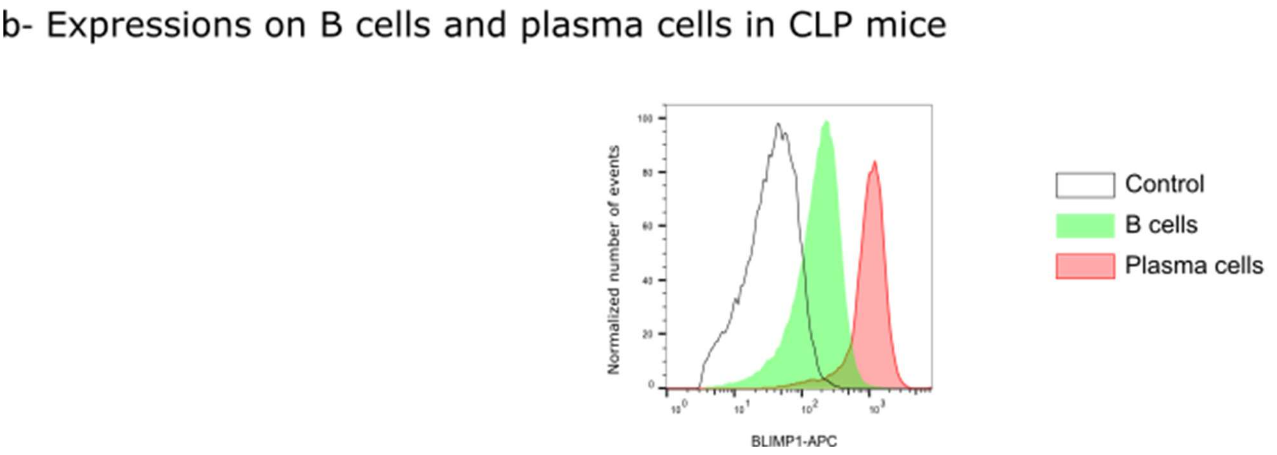

Supplementary Figure 9. Flow cytometry gating strategy and representative examples in healthy donors and patients for cell surface and intracellular stainings.

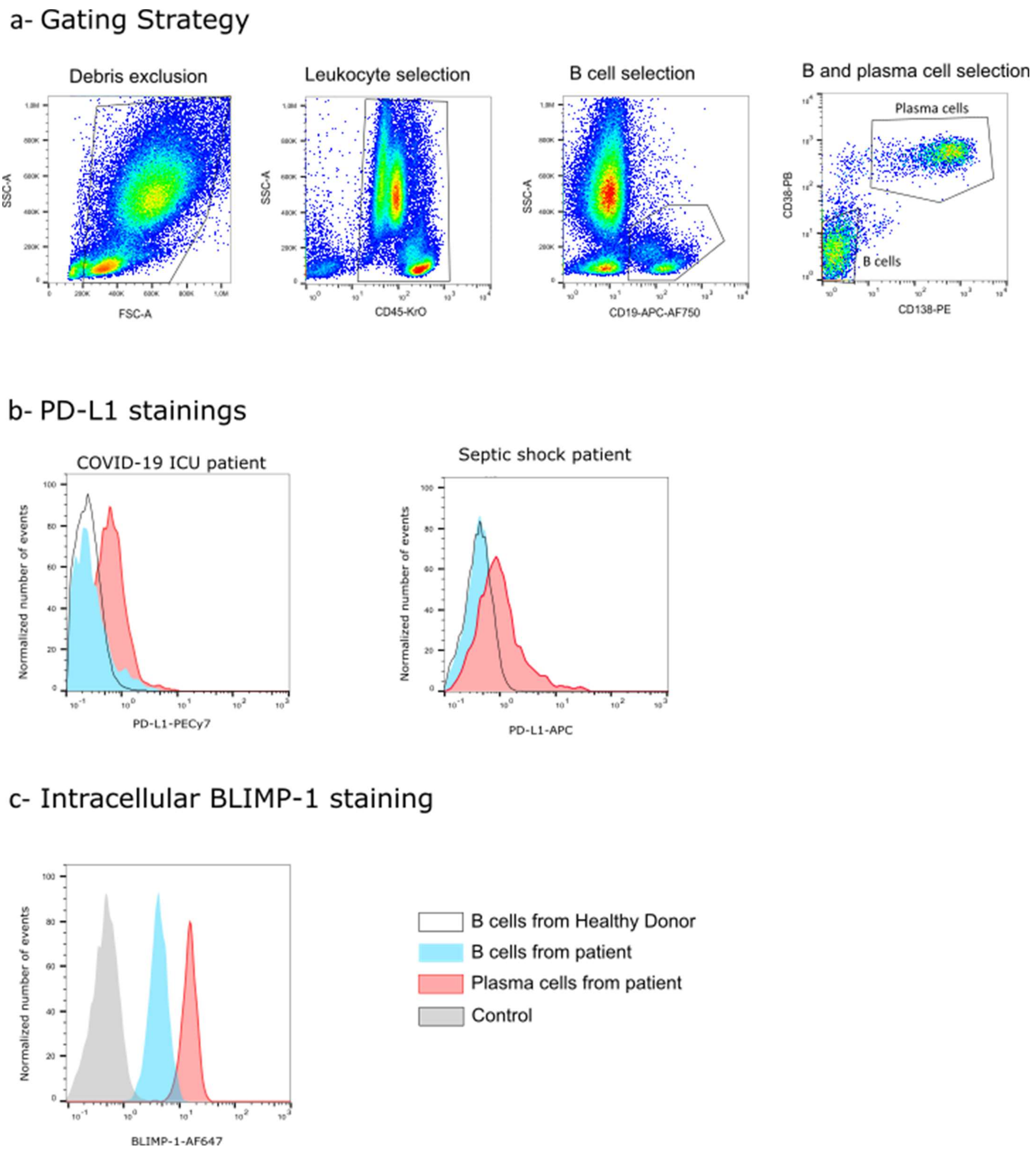

Supplementary Table 1. Demographic and clinical data for critically ill patients with bacterial sepsis and COVID-19. Values are presented as numbers and percentages for categorical variables and medians and [Q1-Q3] interquartiles ranges for continuous variables. SOFA: Sepsis-related organ failure assessment score. SAPS II: simplified acute physiology score. BGP: bacilli Gram negative. CGP: cocci Gram positive. mHLA-DR: MHC class II expression on monocytes. AB/C: numbers of antibodies bound per cells. Normal values = 13 500 – 45 000 AB/C. Age-matched normal values of $CD4^+$ T cell count = 336 – 1126 cell/µL.

	REALISM<br>n= 107	IMMUNOSEPSIS<br>n = 67	RICO<br>n = 33
Type of study	Retrospective	Prospective	Prospective
Age	68 [59 – 77]	72 [61 – 78]	65 [52 – 70]
Gender (Male)	69 (64.5)	42 (63)	25 (76)
MacCabe score			
0	66 (62)	38 (57)	29 (88)
1	34 (32)	27 (40)	3 (9)
2	7 (6)	2 (3)	1 (3)
Charlson score			
0	26 (24)	12 (18)	16 (49)
≥1	81 (76)	55 (82)	17 (52)
SOFA score	8.5 [7 – 11]<br>** 15 missing values*	9 [7 – 11]	3 [2 – 6]
SAPS II score	47 [37 – 55]	54 [42 – 69]	32 [26 – 41]
Infection type	bacterial sepsis	bacterial sepsis	viral sepsis
Infection diagnosis		*1 missing value*	
Microbiology	79 (74)	45 (68)	33 (100)
Radiology	11 (10)	10 (15)	0
Surgery	8 (8)	5 (8)	0
Suspected	9 (8)	6 (9)	0
Site of infection		*3 missing values*	
Pulmonary	29 (28)	12 (19)	33 (100)
Abdominal	43 (41)	27 (42)	0
Other	31 (30)	25 (39)	0
Pathogen type (% among microbiologically proven infections)			
BGN	31 (39)	30 (67)	0
CGP	32 (41)	18 (40)	0
Fungi	10 (13)	3 (7)	0
Virus	2 (3)	0	33 (100)
Other	5 (6)	2 (4)	0
Unidentified	16 (20)	0	0
Mortality at D14	14 (13)	9 (13)	2 (6)
ICU-acquired infections	20 (19)	10 (15)	15 (46)
Immune status at D3			
mHLA-DR (AB/C)	5 565 [3 178 – 9 546]<br>** 19 missing values*	5 012 [3 308 – 8 588]	8 288 [5 868 – 10 808]<br>** 9 missing values*
CD4+ T cells (cells/µL)	397 [280 – 651]<br>** 19 missing values*	350 [197 – 557]	348 [223 – 412]<br>** 9 missing values*

Supplementary Table 2. Severity score criteria for CLP model evaluation

**Fur aspect**	Active grooming	Succinct grooming	Dull coat or bristly hair	Piloerection
**Motor activity**	Normal	Decreased but reactive if stimulated	Decreased even if stimulated	None
**Posture**	Normal	Arched back	Prostrate but free movements	Prostrate motionless, twisted body
**Breathing**	Normal	Normal	Moderate dyspnea	Severe dyspnea
**Weight loss**	0 - 5 %	5 – 10 %	10 – 15 %	> 15 %
**Score**	0	1	2	3

Supplementary Table 3. List of anti-mouse antibodies used in flow cytometry assays

REAGENT or RESOURCE	SOURCE	IDENTIFIER	LOT NUMBER	DILUTION
Rat anti-mouse Blimp-1 (APC) (clone 5E7)	BioLegend	Cat#150008, RRID:AB_2728187	B321722	1:100
Rat anti-mouse CD1d (AF647) (clone 1B1)	BioLegend	Cat#123512, RRID:AB_1236532	B194780	3:100
Armenian Hamster anti-mouse CD3ε (PE) (clone 145-2C11)	BioLegend	Cat#100308, RRID:AB_312673	B376518	1:100
Rat anti-mouse CD3 (BV785) (clone 17A2)	BioLegend	Cat#100231, RRID:AB_11218805	B365876	1:100
Rat anti-mouse CD3 (PE/Dazzle™ 594) (clone 17A2)	BioLegend	Cat#100246, RRID: AB_2565882	B246894	1:100
Rat anti-mouse CD4 (BV421) (clone GK1.5)	BioLegend	Cat#100443, RRID:AB_2562557	B213276; B275080	3:100
Rat anti-mouse CD4 (APC) (clone GK1.5)	BioLegend	Cat#100412, RRID:AB_312696	B372226	1:100
Rat anti-mouse CD4 (BV510) (clone GK1.5)	BioLegend	Cat# 100449, RRID: AB_2564587	B373405	1:100
Rat anti-mouse CD5 (BV421) (clone 53-7.3)	BioLegend	Cat#100617, RRID:AB_2562173	B208111; B213474	3:100
Rat anti-mouse CD8a (PE-Cy7) (clone 53-6.7)	BioLegend	Cat#100722, RRID:AB_312761	B222017	3:100 or 1:100
Rat anti-mouse/human CD11b (BV421) (clone M1/70)	BioLegend	Cat#101251, RRID: AB_11203704	B212710	3:100
Rat anti-mouse/human CD11b (FITC) (clone M1/70)	BioLegend	Cat#101206, RRID: AB_312789	B349919	1:100
Armenian Hamster anti-mouse CD11c (APC) (clone N418)	BioLegend	Cat#117310, RRID: AB_313779	B206713	3:100
Rat anti-mouse CD25 (APC) (clone 3C7)	BioLegend	Cat#101910, RRID:AB_2280288	B218018	3:100
Rat anti-mouse CD40 (PE-Cy7) (clone 3/23)	BioLegend	Cat#124622, RRID:AB_10897812	B226722	3:100
Rat anti-mouse CD44 (BV421) (clone IM7)	BioLegend	Cat#103040, RRID:AB_2616903	B228179	3:100
Rat anti-mouse/human CD44 (BV711) (clone IM7)	BioLegend	Cat#103057, RRID:AB_2564214	B384036	1:100
Rat anti-mouse CD45 (APC-Fire 750) (clone 30-F11)	BioLegend	Cat#103154, RRID:AB_2572116	B226658; B250950; B260280	3:100

Rat anti-mouse CD45 (BV510) (clone 30-F11)	BioLegend	Cat#103138, RRID:AB_2563061	B220123; B220124; B232692; B235434; B240739; B251566; B265567; B280070; B280079; B386738	3:100
Rat anti-mouse/human CD45R/B220 (FITC) (clone RA3-6B2)	BioLegend	Cat#103206, RRID:AB_312991	B230445; B247731	3:100
Rat anti-mouse/human CD45R/B220 (PE-Cy7) (clone RA3-6B2)	BioLegend	Cat#103222, RRID:AB_313005	B246946; B256217	3:100
Rat anti-mouse CD49B (APC) (clone DX5)	BioLegend	Cat#108910, RRID: AB_313417	B375231	1:100
Rat anti-mouse CD62L (BV421) (clone MEL-14)	BioLegend	Cat#104435, RRID:AB_2562560	B372292	1:100
Rat anti-mouse CD69 (PE) (clone H1.2F3)	BioLegend	Cat#104508, RRID:AB_313110	B399727	1:100
Armenian Hamster anti-mouse CD80 (APC) (clone 16-10A1)	BioLegend	Cat#104714, RRID:AB_313135	B227918	3:100
Rat anti-mouse CD86 (BV421) (clone GL-1)	BioLegend	Cat#105031, RRID:AB_10898329	B223811	3:100
Rat anti-mouse CD115 (PE) (clone AFS98)	BioLegend	Cat#135506, RRID:AB_1937253	B190335; B190336	3:100
Rat anti-mouse CD127 (PE) (clone A7R34)	BioLegend	Cat#135010, RRID:AB_1937251	B185924; B185925	3:100
Rat anti-mouse CD138 (APC) (clone 281-2)	BioLegend	Cat#142506, RRID:AB_10962911	B197972; B237677; B239451; B270362;	3:100
Rat anti-mouse CD138 (BV785) (clone 281-2)	BioLegend	Cat#142534, RRID:AB_2814047	B366379	1:100
Rat anti-mouse CD223 (PE) (clone C9B7W)	BioLegend	Cat#125208, RRID:AB_2133343	B247016	3:100
Rat anti-mouse CD267 (PE) (clone 8F10)	BioLegend	Cat#133404, RRID:AB_2240584	B384620	1:100
Rat anti-mouse CD274 (PE) (clone 10F.9G2)	BioLegend	Cat#124307, RRID:AB_2073557	B223286; B246732	3:100
Rat anti-mouse CD274 (APC) (clone 10F.9G2)	BioLegend	Cat#124312, RRID:AB_10612741	B204731; B204732	3:100
Rat anti-mouse CD279 (APC) (clone 29F.1A12)	BioLegend	Cat#135210, RRID:AB_2159183	B412636	1:100

Rat anti-mouse CD279 (BV421) (clone 29F.1A12)	BioLegend	Cat#135217, RRID:AB_10900085	B213656; B226274; B231473; B256184	3:100
Rat anti-mouse CD366 (PE/Dazzle 594) (clone B8.2C12)	BioLegend	Cat#134013, RRID:AB_2632737	B226637; B198857	3:100
Rat anti-mouse F4/80 (PE-Cy7) (clone BM8)	BioLegend	Cat#123114, RRID: AB_893478	B207313	3:100
Rat anti-mouse FoxP3 (FITC) (clone FJK-16s)	eBiosciences	Cat#11-5773-82, RRID:AB_465243	2251241	1:100
Rat anti-mouse IFNγ (FITC) (clone XMG1.2)	BD Biosciences	Cat#554411 RRID: AB_395375	5119572	1:100
Rat anti-mouse I-A/I-E (BV510) (clone M5/114.15.2)	BioLegend	Cat#107635, RRID:AB_2561397	B216155; B244226; B263357; B269686	3:100
Rat anti-mouse IgM (BV510) (clone RMM-1)	BioLegend	Cat#406531, RRID:AB_2650758	B230629; B272542	3:100
Rat anti-mouse IgM (PE-Cy7) (clone RMM-1)	BioLegend	Cat#406513, RRID:AB_10642031	B386037	1:100
Rat anti-mouse Ly6C (PE-Cy7) (clone HK1.4)	BioLegend	Cat#128018, RRID: AB_1732082	B357445	1:100
Rat anti-mouse Ly6G (BV785) (clone 1A8)	BioLegend	Cat#127645, RRID: AB_2566317	B366621	1:100

Supplementary Table 4. List of anti-human antibodies used in flow cytometry assays

REAGENT	SOURCE	IDENTIFIER	LOT NUMBER	DILUTION
Mouse anti-human Blimp-1 (AF647) (clone 6D3)	BD Biosciences	Cat#565002	1096352	1:100
Mouse anti-human CD3 (APC-AF750) (clone UCHT1)	Beckman Coulter	Cat#A94680	200506; 200512	2:100
Mouse anti-human CD4 (APC) (clone 13B8.2)	Beckman Coulter	Cat#IM2468U	200092	2:100
Mouse anti-human CD8 (KrO) (clone B9.11)	Beckman Coulter	Cat#B00067	200047; 200048	2:100
Mouse anti-human CD19 (APC) (clone J3.119)	Beckman Coulter	Cat#IM2470, RRID:AB_130789	200050; 200071; 200072; 200076; 200085; 200096; 200097	5:100
Mouse anti-human CD19 (APC-AF750) (clone J3.119)	Beckman Coulter	Cat#A94681	200064	1:10
Mouse anti-human CD19 (PB) (clone J3-119)	Beckman Coulter	Cat#A86355	200025	5:100
Mouse anti-human CD38 (FITC) (clone HIT2)	BD Biosciences	Cat#555459, RRID:AB_395852	6328698; 8179642; 9140760	5:100
Mouse anti-human CD45 (PB) (clone J33)	Beckman Coulter	Cat#A74763	200044; 200045; 200046; 200048; 200049	5:100
Mouse anti-human CD45 (KrO) (clone J33)	Beckman Coulter	Cat#A96416 RRID: AB_2888654	200110	5:100
Mouse anti-human CD138 (PE) (clone PE)	Beckman Coulter	Cat#A54190	200031; 200037; 200045; 200046	1:10
Mouse anti-human CD274 (PE-Cy7) (PDL1.3.1)	Beckman Coulter	Cat#A78884	200017; 200036; 200038	1:10
Mouse anti-human CD279 (APC) (clone EH12.2H7)	BioLegend	Cat#329908, RRID:AB_940475	B25407; B292213	5:100
Mouse anti-human IgM (RB780) (clone G20-127)	BD Biosciences	Cat#569135	3027595	1:60

Supplementary Table 5. List of biological samples used in methods

SAMPLE TYPE	SOURCE
Patient blood samples	Hospices Civils de Lyon, France
Healthy volunteer blood samples	Etablissement Français du sang, France

Supplementary Table 6. List of experimental mouse models used in methods

STRAIN	SUPPLIER	REFERENCE
C57BL/6J	Charles River	IMSR_JAX: 000664
VertX IL10gfp	The Jackson Laboratory	IMSR_JAX:014530

Supplementary Table 7. List of critical commercial reagent used in methods

REAGENT	SOURCE	IDENTIFIER
Apotracker™ Green	BioLegend	Cat#427402
CellTrace™ Far Red	Thermo Fisher Scientific	Cat#C34564
Click-iT™ EdU Alexa Fluor™ 488 Flow Cytometry Assay Kit	Thermo Fisher Scientific	Cat#C10420
EasySep™ Mouse CD138 Positive Selection Kit	STEMCELL Technologies	Cat#18957
EasySep™ Mouse B Cell Isolation Kit	STEMCELL Technologies	Cat#19854A
EasySep™ Mouse T Cell Isolation Kit	STEMCELL Technologies	Cat#19851A
eBioscience™ FoxP3/transcription factor staining buffer set	Thermo Fisher Scientific	Cat#0055-23
ELISA MAX™ Deluxe Set Mouse IL-10	BioLegend	Cat#431414
Human IFN-gamma DuoSet ELISA	Bio-Techne	Cat#DY285B
MACSprep™ Multiple Myeloma CD138 MicroBeads, human	Miltenyi Biotec	Cat#130-111-744
Mouse IFN-gamma DuoSet ELISA	Bio-Techne	Cat#DY485
nCounter® Mouse PanCancer Immune Profiling Panel	NanoString Technologies	Cat#XT-CSO-MIP1-12
PerFix-nc Kit (no centrifuge assay Kit)	Beckman Coulter	Cat#B31167
Propidium iodide solution	Sigma-Aldrich	Cat#P4864
RNA Lysis Buffer	ZYMO RESEARCH	Cat#R1060-1-100
RNeasy Plus Mini Kit	QIAGEN	Cat#74134
RosetteSep™ Human B Cell Enrichment Cocktail	STEMCELL Technologies	Cat#15024
RosetteSep™ Human T Cell Enrichment Cocktail	STEMCELL Technologies	Cat# 15021
Tag-it Violet™ Proliferation and Cell Tracking Dye	BioLegend	Cat#425101
T Cell Activation/Expansion Kit, mouse	Miltenyi Biotec	Cat#130-093-627
T Cell Activation/Expansion Kit, human	Miltenyi Biotec	Cat#130-091-441
Ultra-LEAF™ Purified anti-mouse CD274 (clone 10F.9G2)	BioLegend	Cat# 124318
Ultra-LEAF™ Purified Rat IgG2b, kappa Isotype Ctrl	BioLegend	Cat# 400644
Zombie Aqua™ Fixable Viability Kit	BioLegend	Cat#423101

Supplementary Table 8. List of chemicals, peptides and recombinant proteins used in methods

REAGENT	SOURCE	IDENTIFIER	LOT NUMBER
Bortezomib	Sigma-Aldrich	Cat#179324-69-7	3187389
Brefeldin A	BioLegend	Cat#420601	B406627
Dimethyl sulfoxyde	Sigma-Aldrich	Cat#D2650	NA
Phytohemagglutinin (PHA)	Thermo Fisher Scientific	Cat#R30852801	3510947
$OVA_{323-339}$/CFA Emulsion	Hooke laboratories	Cat#EK-0132	0106
$OVA_{323-339}$ in tissue culture media	Hooke laboratories	Cat#DS-0141	102-190224F

Supplementary Table 9. List of softwares used in methods

SOFTWARE	SUPPLIER	SOURCE
FlowJo software (version 10.8.1)	BD Biosciences	https://www.flowjo.com/
Gen5 software 3.04	BioTeck	https://www.biotek.com
GraphPad Prism (version 9.0.2)	GraphPad Software	https://www.graphpad.com
Ingenuity Pathway Analysis	QIAGEN	https://digitalinsights.qiagen.com
Kaluza Analysis (version 2.1)	Beckman Coulter	https://www.beckman.com
R (version 3.6.2)	R foundation	https://cran.r-project.org/
