# Supplementary material for: PD-L1+ plasma cells suppress T lymphocyte responses in patients with sepsis and mouse sepsis models
Source: Nat Commun. 2025 Mar 28;16:3030. doi: 10.1038/s41467-025-57706-9 (PMC11953283; doi:10.1038/s41467-025-57706-9)
Supplement: Supplementary file 2 — Reporting Summary [file 41467_2025_57706_MOESM2_ESM.pdf]

nature portfolio

Corresponding author(s): Pr Fabienne VENET

Last updated by author(s): Nov 29, 2023

Reporting Summary

Nature Portfolio wishes to improve the reproducibility of the work that we publish. This form provides structure for consistency and transparency in reporting. For further information on Nature Portfolio policies, see our Editorial Policies and the Editorial Policy Checklist.

Statistics

For all statistical analyses, confirm that the following items are present in the figure legend, table legend, main text, or Methods section.

n/a	Confirmed	
☐	☒	The exact sample size (*n*) for each experimental group/condition, given as a discrete number and unit of measurement
☐	☒	A statement on whether measurements were taken from distinct samples or whether the same sample was measured repeatedly
☐	☒	The statistical test(s) used AND whether they are one- or two-sided<br>*Only common tests should be described solely by name; describe more complex techniques in the Methods section.*
☐	☒	A description of all covariates tested
☐	☒	A description of any assumptions or corrections, such as tests of normality and adjustment for multiple comparisons
☐	☒	A full description of the statistical parameters including central tendency (e.g. means) or other basic estimates (e.g. regression coefficient) AND variation (e.g. standard deviation) or associated estimates of uncertainty (e.g. confidence intervals)
☐	☒	For null hypothesis testing, the test statistic (e.g. *F*, *t*, *r*) with confidence intervals, effect sizes, degrees of freedom and *P* value noted<br>*Give P values as exact values whenever suitable.*
☒	☐	For Bayesian analysis, information on the choice of priors and Markov chain Monte Carlo settings
☐	☒	For hierarchical and complex designs, identification of the appropriate level for tests and full reporting of outcomes
☒	☐	Estimates of effect sizes (e.g. Cohen's *d*, Pearson's *r*), indicating how they were calculated

Our web collection on statistics for biologists contains articles on many of the points above.

Software and code

Policy information about availability of computer code

Data collection	- Kaluza software 2.1 (BECKMAN COULTER)
Data analysis	- Heatmap drawn with pheatmap package in R (version 3.6.2)<br>- Upregulated and downregulated pathways highlighted by Ingenuity Pathway Analysis (QIAGEN)<br>- GraphPad Prism (Version 9.0.2; La Jolla, CA, USA)

For manuscripts utilizing custom algorithms or software that are central to the research but not yet described in published literature, software must be made available to editors and reviewers. We strongly encourage code deposition in a community repository (e.g. GitHub). See the Nature Portfolio guidelines for submitting code & software for further information.

Data

Policy information about availability of data

All manuscripts must include a data availability statement. This statement should provide the following information, where applicable:

- Accession codes, unique identifiers, or web links for publicly available datasets
- A description of any restrictions on data availability
- For clinical datasets or third party data, please ensure that the statement adheres to our policy

Source data are provided with the paper.

Research involving human participants, their data, or biological material

Policy information about studies with human participants or human data. See also policy information about sex, gender (identity/presentation), and sexual orientation and race, ethnicity and racism.

Reporting on sex and gender	Gender is reported for healthy donors and patients included in RICO and IMMUNOSEPSIS4 clinical studies (Supplementary Table S1).
Reporting on race, ethnicity, or other socially relevant groupings	Race, ethnicity or other socially relevant groupings are not reported for healthy donors and patients included in RICO and IMMUNOSEPSIS4 clinical studies as such data cannot be collected in France according to ethic regulation.
Population characteristics	Population characteristics are presented in supplementary Table S1.
Recruitment	Patients were recruited is described in Material and Methods (Pages 21-22).
Ethics oversight	Ethics approval of all observational studies is described in Material and Methods (Pages 21-22).

Note that full information on the approval of the study protocol must also be provided in the manuscript.

Field-specific reporting

Please select the one below that is the best fit for your research. If you are not sure, read the appropriate sections before making your selection.

[x] Life sciences [ ] Behavioural & social sciences [ ] Ecological, evolutionary & environmental sciences

For a reference copy of the document with all sections, see nature.com/documents/nr-reporting-summary-flat.pdf

Life sciences study design

All studies must disclose on these points even when the disclosure is negative.

Sample size	No sample size calculation was performed as this study was purely observational. A minimum of 8 samples obtained from different patients per group was necessary to be able to identify significant differences between groups.
Data exclusions	No data were excluded.
Replication	Data were replicated between 2 cohorts of septic patients and between septic patients and critically ill COVID-19 patients. All attempts at replication were successful.
Randomization	Samples from healthy donors and patients were randomly analyzed during this study. No batch analysis was performed as all experiments were performed with fresh whole blood.
Blinding	The investigators were blinded to group allocation during data collection and/or analysis. Investigators in the lab were blinded of all clinical data related to included patients. In particulier, patients vital status was not known and divulgated to the lab investigator when performing the analyses.

Reporting for specific materials, systems and methods

We require information from authors about some types of materials, experimental systems and methods used in many studies. Here, indicate whether each material, system or method listed is relevant to your study. If you are not sure if a list item applies to your research, read the appropriate section before selecting a response.

Materials & experimental systems

n/a	Involved in the study	
[ ]	[x]	Antibodies
[x]	[ ]	Eukaryotic cell lines
[x]	[ ]	Palaeontology and archaeology
[ ]	[x]	Animals and other organisms
[ ]	[x]	Clinical data
[x]	[ ]	Dual use research of concern
[x]	[ ]	Plants

Methods

n/a	Involved in the study	
[x]	[ ]	ChIP-seq
[ ]	[x]	Flow cytometry
[x]	[ ]	MRI-based neuroimaging

Antibodies

Antibodies used	The list of antibodies, supplier name, catalog number, clone name and lot number are provided in Supplementary Material (Table S3.

	Key Resources Table).
Validation	Only antibodies commercially available and validated by supplier were used in this study. Validation statement can be found in each manufacturer corresponding webpage.

Animals and other research organisms

Policy information about studies involving animals; ARRIVE guidelines recommended for reporting animal research, and Sex and Gender in Research

Laboratory animals	Species, strain and age of animals are reported in Material and Method section (page 21).
Wild animals	No wild animals were used in this study.
Reporting on sex	Only male mice were used in this study to avoid any effect of hormonal cycle on results and because the vast majority of septic patients included in our cohorts are male (< 65 years old - see Table S1).
Field-collected samples	No samples were collected in the field.
Ethics oversight	All the experiments were approved by the local animal ethical evaluation committee (#2016072616404065, CECCAPP, Université Claude Bernard Lyon 1, France) in accordance with European Convention for the Protection of Vertebrate Animals used for Experimental and other Scientific Purposes.

Note that full information on the approval of the study protocol must also be provided in the manuscript.

Clinical data

Policy information about clinical studies

All manuscripts should comply with the ICMJEguidelines for publication of clinical research and a completedCONSORT checklist must be included with all submissions.

Clinical trial registration	Samples in humans were collected under approved protocols registered under ClinicalTrials: NCT04392401 (RICO = REA-IMMUNO-COVID); NCT02638779 (REALISM cohort (REAnimation Low Immune Status Markers); NCT04067674 (IMMUNOSEPSIS 4)
Study protocol	the full study protocol can be obtained from the Principal Investigator of the studies (Pr Thomas Rimmele for REALISM, Pr Fabienne Venet for IMMUNOSEPSIS4 and RICO) upon request.
Data collection	Data were collected between 2019 and 2023 for IMMUNOSEPSIS4 study; between 2020 and 2022 for RICO study and between 2015 and 2018 for REALISM study.
Outcomes	14-days mortality was selected as clinical outcome.

Flow Cytometry

Plots

Confirm that:

- [x] The axis labels state the marker and fluorochrome used (e.g. CD4-FITC).
- [x] The axis scales are clearly visible. Include numbers along axes only for bottom left plot of group (a 'group' is an analysis of identical markers).
- [x] All plots are contour plots with outliers or pseudocolor plots.
- [x] A numerical value for number of cells or percentage (with statistics) is provided.

Methodology

Sample preparation	Sample preparation for murine experiments (spleen) and human samples (whole blood) are described in Material and Methods part (Pages 25-27).
Instrument	Flow cytometry data were acquired on a Navios cytometer (BECKMAN COULTER).
Software	FACS analysis used Kaluza software 2.1 (BECKMAN COULTER).
Cell population abundance	Purity was determined by flow cytometry. Mean purity was 90% for T cells and B cells purified with magnetic beads . It was of 75% for plasma cells purified with magnetic beads. When purified by FACS, plasma cells mean purity was 90%.
Gating strategy	Gating stategy for FCM experiments is shown in Fig S6, Fig S7 and Fig S8.

- [x] Tick this box to confirm that a figure exemplifying the gating strategy is provided in the Supplementary Information.
